# Ureteral transitional cell carcinoma with supraclavicular lymph node metastasis: a case report

**DOI:** 10.1093/jscr/rjad226

**Published:** 2023-04-28

**Authors:** Ankit Gupta, Lavesh Mirpuri, Hussain Hassan, Faizan Malik, Nasira Amtul

**Affiliations:** School of Medicine, University of Leeds, Worsley Building, Woodhouse, Leeds LS2 9JT, UK; School of Medicine, University of Leeds, Worsley Building, Woodhouse, Leeds LS2 9JT, UK; School of Medicine, University of Leeds, Worsley Building, Woodhouse, Leeds LS2 9JT, UK; School of Medicine, University of Leeds, Worsley Building, Woodhouse, Leeds LS2 9JT, UK; Leeds Institute of Emergency General Surgery, St James’s University Hospital, Beckett Street, Leeds LS9 7TF, UK

**Keywords:** Urology, transitional cell carcinoma, urothelial carcinoma

## Abstract

Metastasis to the supraclavicular lymph nodes usually originate from primary tumours in the head and neck, breast or abdomen. Infradiaphragmatic tumours very rarely metastasise to these nodes. Transitional cell carcinomas (TCCs), also termed urothelial carcinomas, account for ⁓90% of all ureteral cancers; exceptionally few cases have reported such cancers spreading to the supraclavicular fossae. We present the case of a 65-year-old male who was being investigated for gallstones and was subsequently found to have metastatic bony lesions and widespread adenopathy on magnetic resonance cholangiopancreatography. Initially, the primary cancer was an area of contention between clinicians, as radiologists suggested it was of urological origin, but the bladder multidisciplinary team felt the scans did not fulfil this notion. Ultimately, histological analysis confirmed the diagnosis of metastatic TCC.

## INTRODUCTION

Ureteral transitional cell carcinoma (TCC) is a type of cancer that affects the lining of the ureter, sharing a common histological subtype and risk factor profile to bladder TCC. Gross or microscopic haematuria is the most common symptom, being present in over 75% of patients [1]. The typical spread of ureteral TCC is to regional lymph nodes such as the iliac, common iliac, external iliac, internal iliac and para-aortic nodes. For patients with more advanced-stage disease or metastatic tumour, sites of node involvement may be more distant. Presence of lymphadenopathy may necessitate more aggressive treatment, such as chemotherapy or radiotherapy, in addition to surgical intervention.

We report an extremely rare case of metastatic ureteral TCC with bilateral supraclavicular node involvement.

## CASE REPORT

**Figure 1:**
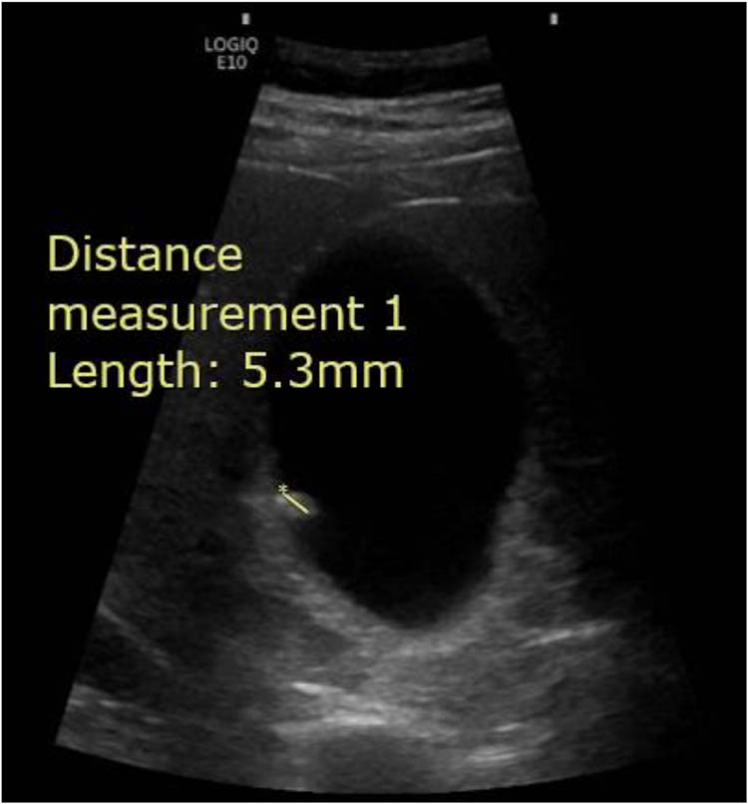
Abdominal ultrasound demonstrating the presence of a 5.3 mm stone within the gallbladder.

A 65-year-old male presented to the Surgical Assessment Unit with a 6-day history of right upper quadrant and epigastric pain. He underwent an ultrasound scan that showed a 5 mm mobile gallstone in a thin-walled non-tender gallbladder and fatty infiltration of the liver (Fig. 1). The biliary ducts were of normal calibre. The patient was given a course of antibiotics and discharged home with a plan to follow-up in the Ambulatory Surgical Clinic in a few weeks’ time. Unfortunately, his pain persisted and had migrated to the right iliac fossa. Apart from the pain, he was otherwise well. He was discharged home with an extended course of antibiotics and a plan to return for a laparoscopic cholecystectomy on the rapid access theatre list.

The patient underwent a pre-operative magnetic resonance cholangiopancreatography (MRCP) that confirmed a solitary 5 mm gallstone within the gallbladder but also picked up various incidental findings. Mild left-sided hydronephrosis, a new bulky left adrenal gland and multiple enlarged para-aortic and abdominal nodes were noted on the scan. Additionally, multifocal bone lesions were identified in the lumbar spine, causing moderate spinal canal narrowing at T9 and T11. Concerns were raised for metastatic spinal cord compression (MSCC) and an urgent magnetic resonance imaging (MRI) scan of the spine was ordered. A primary tumour could not be identified.

**Figure 2:**
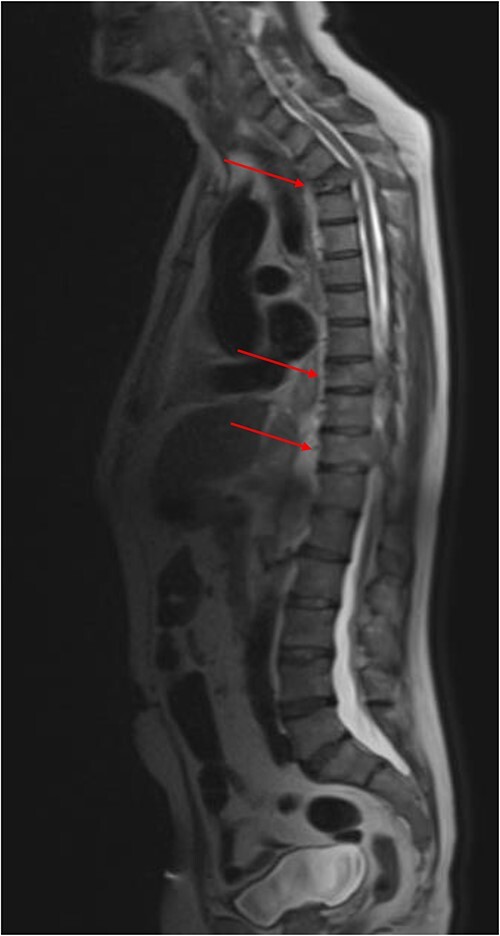
Sagittal MRI whole-spine demonstrating extensive bony metastatic disease. Malignant compromise to the spinal canal at T3, T9 and T11 (red arrows).

MRI revealed extensive bony metastatic disease involving the anterior and posterior elements of the spine and sacrum (Fig. 2). Malignant compromise to the spinal canal at T3, T9 and T11 was noted, as well as extensive malignant adenopathy including the mediastinum and both supraclavicular fossae. Dexamethasone with proton pump inhibitor cover, and palliative radiotherapy to the spine was given for MSCC. Under ultrasound guidance, the left supraclavicular fossa nodes were biopsied and sent for histological analysis.

**Figure 3:**
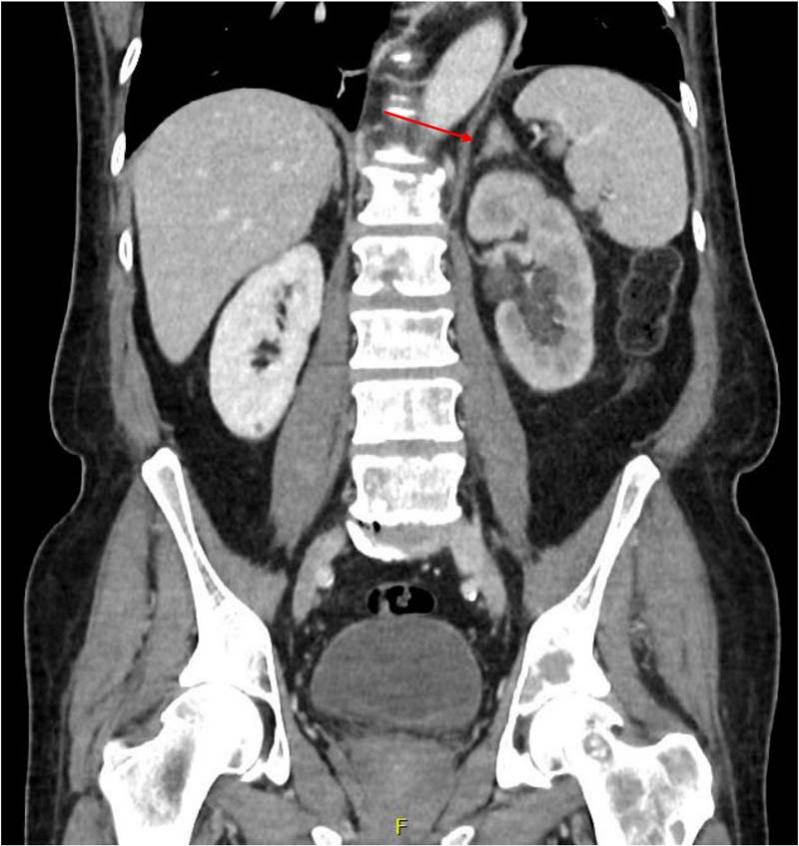
Coronal CT abdomen and pelvis scan with contrast demonstrating left-sided hydronephrosis and left-sided adrenal mass (red arrow).

**Figure 4:**
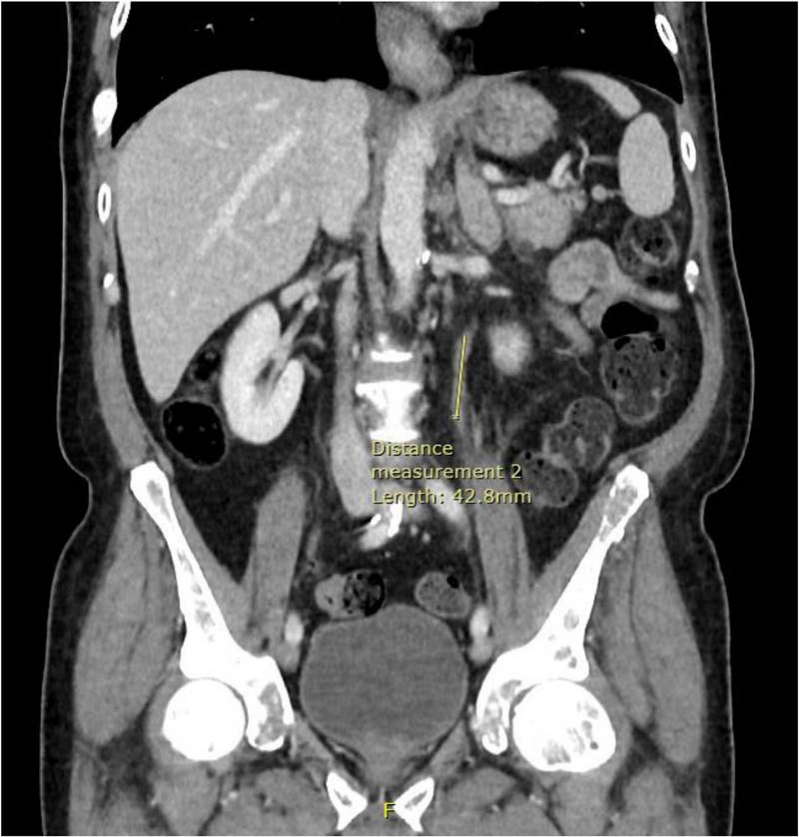
Coronal CT abdomen and pelvis scan with contrast demonstrating urethral enhancement for a distance of 42.8 mm.

Computed tomography (CT) of the thorax, abdomen and pelvis was requested for staging. Lymphadenopathy was present superior and inferior to the diaphragm, left-sided hydronephrosis with reduced enhancement of the left kidney and widespread mixed lytic and sclerotic lesion affecting the skeleton. The bulky left adrenal gland was concerning for adrenal metastasis (Fig. 3). There was also abnormal urothelial enhancement in the left distal ureter for 4 cm, raising the suspicion of urothelial malignancy (Fig. 4). Blood tests showed a normal prostate specific antigen, mild acute kidney injury, CA19–9 of 17 256 and a carcinoembryonic antigen of 18.

Initially the bladder multidisciplinary team felt the CT scan did not provide sufficient evidence of a TCC due to the changes being non-specific. Ultimately, however, the pathologists reviewed the biopsied specimens and concluded that it had very typical characteristics and immunohistochemical appearance for TCC. The patient was embarked upon a palliative care pathway due to the extent of the disease.

Unfortunately, 3 weeks later, the patient was acutely admitted to hospital with shortness of breath and a fall with a long lie. His neck had been tilted to the right side with head droop for the preceding 4 days, likely due to progression of MSCC to the cervical spine. He was found to be COVID-19 positive on admission and sadly died on day 3 of his admission.

## DISCUSSION

TCC is the most common histological type of ureteral carcinoma, accounting for ⁓90% of ureteral cancers. The most frequently affected sites of nodal involvement are the obturator and internal iliac regions [2]. Here, we present an extremely rare case of malignant TCC with supraclavicular node metastasis, found incidentally following bony lesions and widespread adenopathy on MRCP.

To our knowledge, only two case reports of ureteral TCC with supraclavicular node metastasis have been reported [3, 4]. Moreover, a further four cases have been published of bladder TCC with supraclavicular node metastasis. Two cases presented with generalised lymphadenopathy [5, 6], one with isolated supraclavicular node metastasis alongside regional node metastasis [7], and one with cervical lymph node metastasis without generalised lymphadenopathy [8]. These reports, alongside ours, demonstrate that the pattern of head and neck involvement in TCC is highly heterogenous.

Serum CA-19-9 has been found to be almost invariably raised in patients with metastatic urothelial carcinoma [9]. Studies have found that increased serum or urinary CA19–9 may reflect poor prognosis of a urinary system tumour [10]. Changes in serum CA19–9 may also be indicative of the progression or regression of disease and may help identify recurrence due to a lead time of 4–6 months before the clinical diagnosis is made [11, 12].

The rarity of our case conveys important messages to clinicians. A lack of clinical clues makes even the suspicion of TCC challenging, let alone a definitive diagnosis. Patients may lack the classical symptoms and signs that accompany TCC and may present late in their disease process. As with our case, even if the patient presented with a different clinical issue, incidental findings should be acted upon promptly to ensure as optimal a clinical outcome as possible.

To conclude, TCC is the most common neoplasm of the urinary system. Supraclavicular lymphadenopathy may be classically recognised in association with upper body cancers; clinicians should keep a high index of suspicion for cancers originating from the pelvis. Whilst extremely rare, we hope to use our case to add to the body of reports detailing the atypical nodal spread of TCC.
